# A comparison of stiffness of six knee braces with application for posterolateral corner reconstructions

**DOI:** 10.1177/09544119231188009

**Published:** 2023-07-21

**Authors:** Kirsten Hickie, Yuelin Qiu, Jonelle Jn Baptiste, Andrew Salipas, Stephanie Nathanail, Lindsey Westover, Mark Sommerfeldt

**Affiliations:** 1 Division of Orthopaedic Surgery, Department of Surgery, Faculty of Medicine and Dentistry, University of Alberta, Edmonton, AB, Canada; 2 Department of Civil and Environmental Engineering, Faculty of Engineering, University of Alberta, Edmonton, AB, Canada; 3 Orthopaedic Surgery, Alberta Health Services, Edmonton, AB, Canada; 4 Department of Mechanical Engineering, Faculty of Engineering, University of Alberta, Edmonton, AB, Canada

**Keywords:** Biomechanical testing/analysis, biodynamics, biomaterials stress analysis, knee biomechanics, orthopedic materials, rehabilitation devices

## Abstract

Posterolateral corner knee injuries are clinically significant, and often require surgical reconstruction. The optimal knee brace following posterolateral corner reconstructions has not yet been determined via clinical nor biomechanical study. We sought to evaluate the stiffness of six types of knee braces to determine the ideal brace type for reducing varus forces, which may have clinical utility for posterolateral corner knee reconstruction rehabilitation. Six different groups of knee braces underwent mechanical testing: cruciate braces, cruciate braces with a valgus bend, medial unloaders, articulating sleeves, hinged braces, and tri-panel immobilizers. Each brace was fitted to the same fiberglass leg model and was secured to the testing apparatus. Force was applied under four-point bending to generate a varus moment about the artificial knee. The stiffness in Newtons per millimeter (N/mm) of each brace was calculated from the slope of the force-displacement curve. The cruciate brace with a valgus bend had the highest average stiffness at 192.61 N/mm (SD 28.53). The articulating sleeve was the least stiff with an average stiffness of 49.86 N/mm (SD 8.99). Stiffness of the cruciate brace was not statistically different compared to cruciate valgus (*p* = 0.083) or medial unloader (*p* = 0.098). In this experimental design, a cruciate brace with a valgus bend was shown to have the highest overall stiffness, while an articulating sleeve had the lowest stiffness. Future work will investigate whether this translates into clinical performance.

## Introduction

Posterolateral corner (PLC) knee injuries are clinically important traumatic events that if missed, can have debilitating consequences on patient outcomes.^1–3^ These injuries most commonly occur due to a high energy mechanism (e.g. football injury, motorcycle accident), with combined hyperextension and varus force. The resultant damage to the soft-tissue structures of the posterolateral corner of the knee can lead to increased laxity of those lateral structures, a varus thrust, and early post-traumatic arthritis of the knee. This can lead to difficulty with return to sports and high-level activity, as well as stairs and other activities of daily living. Multiple studies support the operative repair or reconstruction of severe grade III PLC injuries that occur with associated functional instability.^1–5^ However, even with surgical management, residual varus laxity has been found in up to 19% of posterolateral corner injuries, and graft failure rates can be as high as 23%.^6^ Consensus data promotes the use of knee braces postoperatively to protect the reconstruction or repair during rehabilitation; however, no known clinical studies have investigated the superiority of a specific type of brace for postoperative immobilization.^1,7^ Further, it is unknown as to the brace type that best withstands varus force when tested in a controlled laboratory setting. Considering the lack of clinical and mechanical evidence, surgeons and rehabilitation professionals face uncertainty regarding the most beneficial brace to recommend to their patients’ following PLC reconstruction.

There are a variety of immobilizing knee braces available, including cruciate-protecting braces, medial unloader, hinged knee sleeves, hinged braces, and fixed extension splints (also known as tri-panel immobilizers). Biomechanical studies support the use of valgus knee braces for unloading in medial compartment osteoarthritis.^8^ Medial unloading braces work through multiple mechanisms to decrease the force experienced by the medial compartment of the knee. In essence, they strut the medial side of the knee, holding the knee in a valgus position, preventing the knee from drifting into varus^8^; given their ability to decrease the load experienced by the medial compartment articular surfaces, and in doing so, generate a more valgus orientation of the knee joint, we postulate that this type of brace may concurrently prevent tension from developing in the lateral knee soft-tissue structures, including posterolateral corner structures. Posterolateral corner injuries may occur in association with other ligamentous knee injuries, such as cruciate injuries. The medial unloader brace might work in isolation to protect the lateral knee structures, while a cruciate brace that is bent into valgus could protect both the posterolateral corner as well as the cruciate injury. Medial unloading or cruciate-protecting knee braces bent into valgus may be superior at protecting the posterolateral corner of the knee following reconstruction or repair when compared to other brace types. Given that no known study has shown superiority of one brace type over another, we elected to test multiple different knee braces, including the types discussed here in detail. To address the paucity of evidence regarding the mechanical properties of knee braces currently in use in the setting of postoperative posterolateral corner injuries, this mechanical study sought to determine the stiffness of each brace type in a laboratory setting.

## Methods

**Figure 1.**
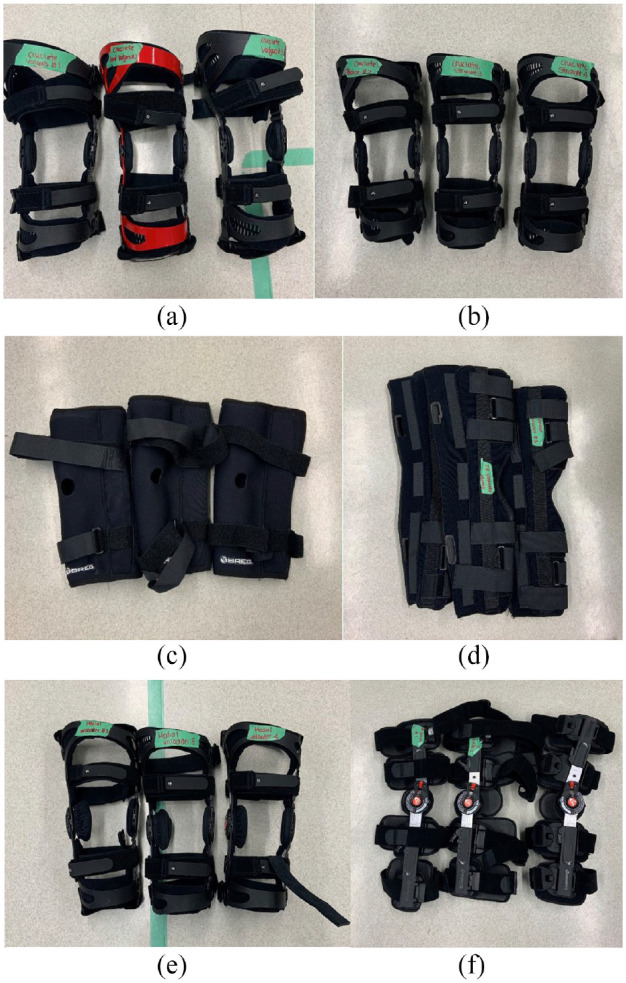
The six knee braces utilized: (a) cruciate valgus, (b) cruciate straight, (c) articulating sleeve, (d) tri-panel immobilizer, (e) medial unloader, and (f) hinge knee brace.

This study investigated six brace types with 3–4 samples in each group. This was based on availability of each brace from our supplier: cruciate (*n* = 3), cruciate with a valgus bend (*n* = 3), medial unloader (*n* = 4), hinged (*n* = 3), articulating sleeve (*n* = 3), tri-panel immobilizer (*n* = 3) (Figure 1). The make of the braces used for this study were Breg (Breg, Canada). The cruciate knee brace contains a semi-rigid articulating hinge that allows full sagittal motion of the knee while resisting varus and valgus forces and anterior tibial translation. The valgus cruciate brace was a cruciate brace that was pre-contoured into slight valgus to further resist varus force through the knee. As previously described, the medial unloader brace struts the medial side to offload the medial compartment of the knee. The hinge knee brace provides purely coronal plane stability while allowing full flexion and extension. The articulating sleeve is the least constrained brace that consists of a circumferential neoprene supportive orthosis. Finally, the tri-panel immobilizer contains two rigid struts along the medial and lateral aspects of the knee with straps that hold the knee in a fixed position of full extension.

A five-layered fiberglass leg model filled with light-weight rigid foam was constructed to represent the average leg size and allow for the braces to be tested in the configuration that they would typically be worn. The leg model was created by a local orthotist, who made the model to represent the leg circumference of an average-sized 70 kg adult based on their expertise. A 1-inch segment was cut out circumferentially at the level of the knee to allow for six planes of movement about the knee of the model (flexion and extension; varus and valgus; internal and external rotation) and thus isolate the stiffness of the brace. The purpose of the leg model itself was simply to allow for the brace to be filled and strapped in its real-life configuration. Multiple trials were run on each individual brace. After each trial, the brace was removed and re-strapped onto the leg model before running the next trial. This was performed by a single person to ensure consistency in application of the braces to the leg model, and the same leg model was used for each trial. A total of 69 trials were run (Table 1).

**Table 1.** Number of test trials by brace type and sample number.

Sample #	Number of trials					
	Cruciate straight	Cruciate valgus	Medial unloader	Sleeve with hinge	Hinged	Tri-panel immobilizer
1	4	4	4	3	3	4
2	4	4	3	4	3	4
3	4	4	4	3	3	4
4	–	–	3	–	–	–

**Figure 2.**
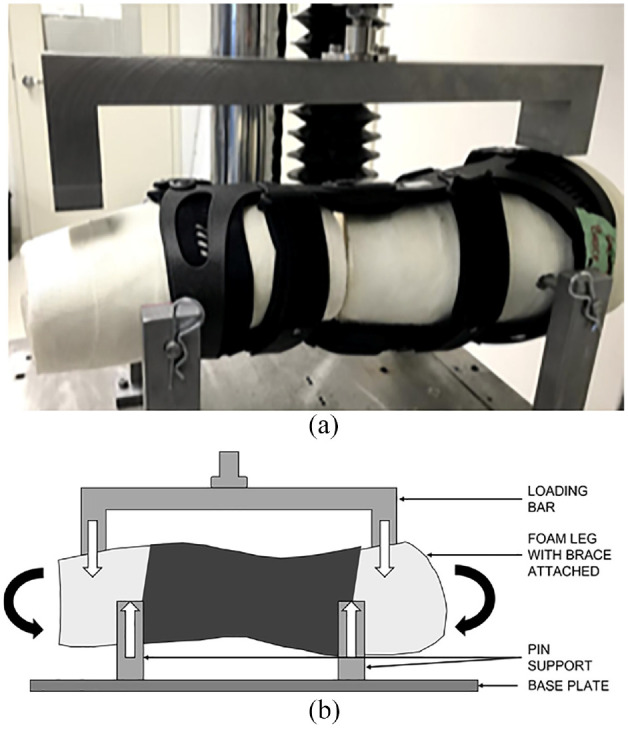
(a) Lower limb model with medial unloader brace loaded on the testing apparatus and (b) pictural representation of lower limb model with medial unloader brace during testing. Downward white arrows represent applied load from the test instrument. Upward white arrows represent the reaction forces from the pin supports. Black arrows represent the moment applied to the leg model created by the loading, which will be resisted by the brace.

Each brace was fitted to the leg model and tested using a Bose ElectroForce 3510 mechanical test instrument (TA Instruments). The leg model was oriented horizontally in the test frame with the lateral side pointing upwards. The thigh and leg portions of the lower limb model were fixed to the bottom platform of the mechanical testing frame using pin supports to allow for free varus/valgus rotation (Figure 2). A horizontal metal bar was fixed to the top loading arm of the mechanical test instrument to apply a downward load to the proximal (thigh) and distal (ankle) portions of the leg model under four-point bending. Three-dimensionally printed forks were attached to the contacting ends of the loading bar to ensure proper contact with the leg model. A 20 N preload was applied to establish contact and to account for compression of foam within the brace. Subsequently, a load was applied through the bar at a rate of 0.1 mm/s to a total displacement of 7 mm (displacement control) to generate a varus moment about the knee. Linear displacement was recorded in millimeters and the applied force was measured in Newtons using the mechanical test instrument. The stiffness of each brace was calculated from the slope of the force-displacement curve. The overall stiffness was determined from the slope of the force-displacement curve between 2 and 5 mm of displacement. Statistical analysis was performed using JASP (JASP team 2020). Average and standard deviation (SD) values were determined from all tests of all samples of each brace type. ANOVA with linear contrasts assuming unequal variance was used to compare stiffness values between brace types looking at overall stiffness. Standard statistical analyses were used with *p* < 0.05 considered statistically significant when comparative analysis was performed.

## Results

**Figure 3.**
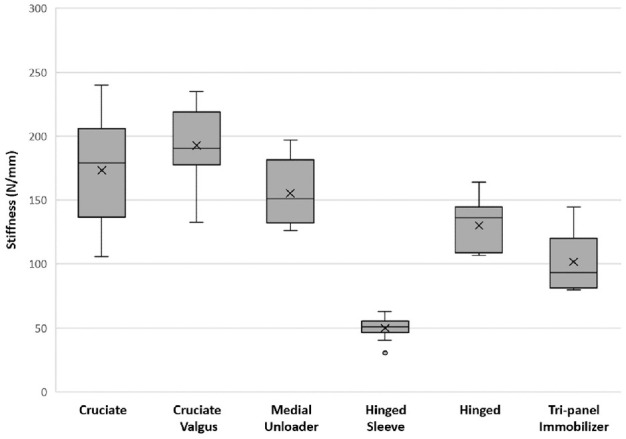
Average overall stiffness for the different brace types.

Figure 3 depicts the overall average stiffness during 2–5 mm of displacement for each brace type during testing. The brace type with the highest average stiffness was the cruciate valgus brace at (192.61 ± 28.53 N/mm), followed by the cruciate brace (173.19 ± 42.44 N/mm), the medial unloader brace (155.33 ± 24.33 N/mm), hinged brace (130.17 ± 20.76 N/mm), tri-panel immobilizer (101.69 ± 22.11 N/mm), and the hinged sleeve brace (49.86 ± 8.99 N/mm).

When compared to all the other brace types except for the cruciate type, the cruciate valgus brace was the stiffest and this was statistically significant (Table 2). The average stiffness of the cruciate brace was not found to be significantly different when compared to that of the cruciate brace with a valgus bend (*p* = 0.204), and the medial unloader brace (*p* = 0.215). All other comparisons between each brace type were found to be statistically significant (*p* < 0.05). This is summarized in Table 2.

**Table 2.** *p*-Values for brace type comparisons of overall stiffness. Statistical significance is represented by *.

	Cruciate	Cruciate valgus	Medial unloader	Hinged sleeve	Hinged	Tri-panel immobilizer
Cruciate		*p* = 0.204	*p* = 0.215	*p < 0.001	*p = 0.007	*p < 0.001
Cruciate valgus			*p = 0.002	*p < 0.001	*p < 0.001	*p < 0.001
Medial unloader				*p < 0.001	*p = 0.016	*p < 0.001
Hinged sleeve					*p < 0.001	*p < 0.001
Hinged						*p = 0.007
Tri-panel immobilizer						

## Discussion

The most important finding of this study is that in a controlled laboratory setting, stiffness against varus force is significantly higher with a cruciate brace with a valgus bend, cruciate brace, and medial unloader brace, as compared to a hinged brace, articulating sleeve brace, and tri-panel immobilizing brace. These findings demonstrate the mechanical properties of common postoperative knee braces and provide a framework for further clinical research, with the goal to ultimately improve patients’ surgical outcomes after posterolateral corner repair or reconstruction.

Posterolateral corner injuries are extremely challenging to treat due to significant injury to the complex supporting structures of the knee. Residual varus laxity can be seen in up to 19% of PLC reconstructions and this can significantly affect functional outcomes, leading to varus thrust gait and osteoarthritis.^7^ Accordingly, focus has been placed on improving the ability to accurately diagnose and effectively treat these injuries.

There is a paucity of evidence in the literature regarding optimal postoperative management pertaining to posterolateral corner reconstruction; further, even professional consensus is lacking. In a survey of experts, heterogeneity in approaches to treatment and rehabilitation of posterolateral corner injuries was highlighted.^1^ While 96.4% of respondents agreed that a brace should be used following PLC surgery, no consensus was elicited regarding which specific type of brace should be employed, and most often, the recommendation is simply left as “knee immobilizer.”

Bracing after knee surgery in general remains a significantly debated topic. A recent systematic review of studies that investigated brace use after anterior cruciate ligament reconstruction (ACLR) found that bracing may impart some mechanical benefit but that bracing in and of itself likely does not decrease the rate of reinjury.^9^ A more recent systematic review similarly found that the effectiveness of bracing at time of return to sport remains ambiguous.^10^ Bracing after posterior cruciate ligament reconstruction (PCLR) however, is a commonly agreed upon treatment recommendation. In a review of published PCLR rehabilitation protocols, 42 of 44 protocols advocated for immediate and prolonged bracing after surgery.^11^ It is generally accepted that bracing after PCL reconstruction may lessen forces through the reconstructed ligament, especially forces that increase with increasing knee flexion.^11^ It is evident that recommendations for bracing after PLC reconstruction are more in keeping with recommendations for bracing after PCLR than after ACLR. This likely reflects the experienced clinician’s belief that any varus moment through the knee will put stress on the reconstructed PLC structures and may lead to residual varus laxity.

The implications of residual varus laxity are significant. Functional instability, pain, and increased long-term risk of osteoarthritis have been demonstrated in this scenario.^12^ Clinicians agree that chronic varus laxity can be challenging to address, and might include revision reconstruction and/or valgus osteotomy; these are complicated procedures associated with substantial recovery time, cost, and risk of complications for the patient. Given the high incidence of postoperative varus laxity after PLC reconstruction, bracing may be a treatment tool where benefit can be imparted.

The ability of knee braces to alter mechanics of the knee are well established. Medial unloader braces have been shown to lessen contact forces through the medial compartment by holding the knee in a valgus position.^13^ A similar type of brace may be able to offer protection to the structures of the PLC after reconstruction, and our study lays the groundwork for clinical work in this area. Further investigation with a cadaveric model and clinical study is necessary to ultimately determine the utility of a stiffer brace for protection of the PLC reconstruction from varus forces in vivo.

It is important to keep in mind that there are potential consequences to brace use after surgery. Stiffness, muscle atrophy, and thromboembolic disease are all issues that may be affected by brace use.^14,15^ Increased case cost is another factor that all clinicians should weigh as they make recommendations to their patients. A hinged knee brace can be purchased for between $100 and 300 USD, whereas a custom cruciate brace with or without a valgus bend may require special referral and cost upwards of several thousand dollars, which may be prohibitive for some patients. However, if varus laxity can be mitigated by bracing, future overall healthcare expenditures could potentially be lessened. Further research is required before specific bracing recommendations, including duration of postoperative use, can be made and before cost-effectiveness can be demonstrated.

We recognize several study limitations. First, the sample sizes for each brace were small. We attempted to account for this by running multiple tests on each brace to increase the overall number of tests completed. The number of braces per type varied depending on the availability from our supplier. Furthermore, for each brace, only a single brand was used which may decrease the generalizability to other brace brands. Additionally, the braces were only stressed in varus in fixed extension which does not represent in vivo use, where braces would undergo full range of motion after the acute postoperative period. Similarly, there was no rotational or axial component to the stress applied to the braces, and therefore, we were unable to account for real-world scenarios involving alternative forces applied to the brace. Finally, as per the mechanical nature of the study, the clinical significance of the improved stiffness of the cruciate valgus brace and its role in posterolateral corner reconstruction rehabilitation is yet unknown.

## Conclusions

In this study, we have demonstrated a statistically significant difference in the stiffness of different knee brace types that could be used for protecting posterolateral corner repairs or reconstructions. Stiffness against varus force was found to be significantly higher with a cruciate brace with a valgus bend, cruciate brace, and medial unloader brace, as compared to a hinged brace, articulating sleeve brace, and tri-panel immobilizing brace. Further research is needed to ascertain how the results of this study translate into clinical practice.

## References

[1] Chahla J Murray IR Robinson J , et al. Posterolateral corner of the knee: an expert consensus statement on diagnosis, classification, treatment, and rehabilitation. Knee Surg Sports Traumatol Arthrosc 2019; 27(8): 2520–2529. 30478468 10.1007/s00167-018-5260-4

[2] Cooper JM McAndrews PT LaPrade RF . Posterolateral corner injuries of the knee: anatomy, diagnosis, and treatment. Sports Med Arthrosc Rev 2006; 14(4): 213–220. 17135971 10.1097/01.jsa.0000212324.46430.60

[3] Levy BA Stuart MJ Whelan DB . Posterolateral instability of the knee: evaluation, treatment, results. Sports Med Arthrosc Rev 2010; 18(4): 254–262. 21079505 10.1097/JSA.0b013e3181f88527

[4] Chahla J Moatshe G Dean CS , et al. Posterolateral corner of the knee: current concepts. Arch Bone Jt Surg 2016; 4(2): 97–103. 27200384 PMC4852053

[5] Levy BA Boyd JL Stuart MJ . Surgical treatment of acute and chronic anterior and posterior cruciate ligament and lateral side injuries of the knee. Sports Med Arthrosc Rev 2011; 19(2): 110–119. 21540708 10.1097/JSA.0b013e3182191c75

[6] Geeslin AG Moulton SG LaPrade RF . A systematic review of the outcomes of posterolateral corner knee injuries, part 1: surgical treatment of acute injuries. Am J Sports Med 2016; 44(5): 1336–1342. 26260464 10.1177/0363546515592828

[7] Lynch AD Chmielewski T Bailey L , et al. Current concepts and controversies in rehabilitation after surgery for multiple ligament knee injury. Curr Rev Musculoskelet Med 2017; 10(3): 328–345. 28779476 10.1007/s12178-017-9425-4 PMC5577426

[8] Moyer RF Birmingham TB Bryant DM , et al. Biomechanical effects of valgus knee bracing: a systematic review and meta-analysis. Osteoarthr Cartil 2015; 23(2): 178–188. 10.1016/j.joca.2014.11.018 25447975

[9] Lowe WR Warth RJ Davis EP , et al. Functional bracing after anterior cruciate ligament reconstruction: a systematic review. J Am Acad Orthop Surg 2017; 25(3): 239–249. 28195986 10.5435/JAAOS-D-15-00710

[10] Marois B Tan XW Pauyo T , et al. Can a knee brace prevent ACL reinjury: a systematic review. Int J Environ Res Public Health 2021; 18(14): 7611–7622. 34300065 10.3390/ijerph18147611 PMC8303933

[11] Senese M Greenberg E Todd Lawrence J , et al. Rehabilitation following isolated posterior cruciate ligament reconstruction: a literature review of published protocols. Int J Sports Phys Ther 2018; 13(4): 737–751. 30140567 PMC6088114

[12] Kannus P . Nonoperative treatment of grade II and III sprains of the lateral ligament compartment of the knee. Am J Sports Med 1989; 17(1): 83–88. 2929843 10.1177/036354658901700114

[13] Self BP Greenwald RM Pflaster DS . A biomechanical analysis of a medial unloading brace for osteoarthritis in the knee. Arthritis Care Res 2000; 13(4): 191–197. 14635273 10.1002/1529-0131(200008)13:4<191::aid-anr3>3.0.co;2-c

[14] Kannus P . Immobilization or early mobilization after an acute soft-tissue injury? Phys Sportsmed 2000; 28(3): 55–63. 20086628 10.3810/psm.2000.03.775

[15] Sommerfeldt M Bouliane M Otto D , et al. The use of early immobilization in the management of acute soft-tissue injuries of the knee: results of a survey of emergency physicians, sports medicine physicians and orthopedic surgeons. Can J Surg 2015; 58(1): 48–53. 25621910 10.1503/cjs.004014 PMC4309764

